# Comparison of a Hybrid IMRT/VMAT technique with non-coplanar VMAT and non-coplanar IMRT for unresectable olfactory neuroblastoma using the RayStation treatment planning system—EUD, NTCP and planning study

**DOI:** 10.1093/jrr/rrab010

**Published:** 2021-04-12

**Authors:** Vijay P Raturi, Atsushi Motegi, Sadamoto Zenda, Naoki Nakamura, Hidehiro Hojo, Shin-Ichiro Kageyama, Masayuki Okumura, Toshiya Rachi, Hajime Ohyoshi, Hidenobu Tachibana, Kana Motegi, Takaki Ariji, Masaki Nakamura, Yasuhiro Hirano, Hidenari Hirata, Tetsuo Akimoto

**Affiliations:** 1 Division of Radiation Oncology and Particle Therapy, National Cancer Center Hospital East, Kashiwa, Chiba, Japan; 2 Course of Advanced Clinical Research of Cancer, Graduate School of Medicine, Juntendo University, Tokyo, Japan; 3 Department of Radiology, St. Marianna University School of Medicine, Kawasaki, Japan

**Keywords:** olfactory neuroblastoma (ONB), intensity-modulated radiotherapy (IMRT), volumetric-modulated radiotherapy (VMAT), normal tissue complication probability (NTCP), equivalent uniform dose (EUD)

## Abstract

The purpose of this study was to compare hybrid intensity-modulated radiotherapy (IMRT) and volumetric-modulated arc therapy (Hybrid IMRT/VMAT), with non-coplanar (nc) IMRT and nc-VMAT treatment plans for unresectable olfactory neuroblastoma (ONB). Hybrid IMRT/VMAT, nc-IMRT and nc-VMAT plans were optimized for 12 patients with modified Kadish C stage ONB. Dose prescription was 65 Gy in 26 fractions. Dose–volume histogram parameters, conformation number (CN), homogeneity index (HI), integral dose and monitor units (MUs) delivered per fraction were assessed. Equivalent uniform dose (EUD) and normal tissue complication probability (NTCP) based on the EUD model (NTCP_Logit_) and the Lyman–Kutcher–Burman model (NTCP_LKB_) were also evaluated. We found that the Hybrid IMRT/VMAT plan significantly improved the CN for clinical target volume (CTV) and planning treatment volume (PTV) compared with the nc-VMAT plan. In general, sparing of organs at risk (OARs) is similar with the three techniques, although the Hybrid IMRT/VMAT plan resulted in a significantly reduced D_max_ to contralateral (C/L) optic nerve compared with the nc-IMRT plan. The Hybrid IMRT/VMAT plan significantly reduce EUD to the ipsilateral (I/L) and C/L optic nerve in comparison with the nc-IMRT plan and nc-VMAT plan, but the difference in NTCP between the three technique was <1%. We concluded that the Hybrid IMRT/VMAT technique can offer improvement in terms of target conformity and EUD for optic nerves, while achieving equal or better OAR sparing compared with nc-IMRT and nc-VMAT, and can be a viable radiation technique for treating unresectable ONB. However, the clinical benefit of these small differences in dosimetric data, EUD and NTCP of optic nerves may be minimal.

## INTRODUCTION

Sinonasal cancers account for ~3–5% of all head and neck cancers [1]. Investigators have estimated that olfactory neuroblastomas (ONBs) constitute 3–6% of all sinonasal cancers, and the incidence of ONB seems to have increased in the last decade [2]. ONBs are thought to arise from the specialized sensory neuroepithelial olfactory epithelium within the upper region of the nasal cavity. The most widely used ONB staging system is the revised Kadish system, and at the time of diagnosis most tumors are stage C (~50%) [3].

One of the most characteristic imaging findings of ONB is a ‘dumbbell-shaped’ mass extending across the cribriform plate, and extension to and erosion of the cribriform plate occurs during the early stage of the disease [4]. Involvement of the cribriform plate is an important prognostic factor for ONB, and it is significantly associated with poorer local control and overall survival [5, 6]. Involvement of the cribriform plate also brings the tumor into close proximity with normal tissues and organs, including the brainstem and optic chiasm. No effective treatment modalities have been established for advanced ONB. Complete resection is challenging in the advanced stage, and multimodality treatment has been shown to be more effective [7–9]. During radiation therapy for ONB, it is quite challenging to achieve optimal radiation dose coverage for planning target volume (PTV), because critical neurological and orbital organs surround it, and in many patients the PTV is close to optic pathway structures, the brainstem and other parts of the brain.

Numerous studies have explored radiotherapy techniques including particle therapy for use in the treatment of tumors in the nasal cavity and paranasal sinus tumors [8, 9]. The results of a study by Huang *et al.* showed that intensity-modulated radiotherapy (IMRT) plans produce better tumor coverage and sparing of organs at risk (OARs) compared with three-dimensional conformal radiotherapy (3D-CRT) [10]. The use of non-coplanar (nc) beams in IMRT and volumetric-modulated arc therapy (VMAT) plans may provide additional optimization freedom within the inverse planning process for target volume coverage and OAR sparing [11, 12]. The studies by Orlandi *et al.* and Jeong *et al*. that compared coplanar and nc-VMAT versus co-planar IMRT and co-planar VMAT versus nc-IMRT showed that nc-VMAT provided better conformity and OAR sparing with fewer delivered monitor units (MUs) and less treatment time than did IMRT [13, 14]. A Hybrid IMRT/VMAT technique using a simultaneously optimized algorithm combining IMRT with desired intensity modulation and VMAT with desired angular beam sampling can improve dose distribution so that the full potential of the hybrid technique may be exploited.

The validation of radiotherapy plans is normally based upon the dosimetric information using a cumulative dose–volume histogram (DVH) and 3D anatomical dose distribution that may not always correlate with the clinical outcome. While taking the medical decision to select the best treatment plan, in addition to dosimetric parameters, a robust surrogate of dose distribution such as normal tissue complication probability (NTCP) and equivalent uniform dose (EUD) should also be assessed as a radiobiological guide, as it evaluates the treatment plan by analyzing the entire DVH [15, 16].

The purpose of this study is to identify, based on radiobiological and planning parameters, the efficacy of a proposed Hybrid IMRT/VMAT technique in comparison with nc-IMRT and nc-VMAT, in improving the therapeutic ratio and sparing OARs in the treatment of unresectable ONB.

## MATERIALS AND METHODS

Patient data in the hospital medical records were reviewed. After receiving approval for this study, from the Institutional Research Ethics Committee reference number: 2010R006), 12 patients with modified Kadish stage C, biopsy-confirmed ONB between 2011 and 2019 were identified. The 1 mm slice thickness planning computed tomography (CT) images of the 12 patients were retrieved.

### Target volume and OAR delineation

Planning CT images were fused with magnetic resonance imaging (MRI; T1 and T2) images for contouring, as shown in Supplementary Fig. 1. Gross disease was delineated as gross tumor volume (GTV). The clinical target volume (CTV) included the GTV and adjacent sinus. For the PTV, CTV was expanded with an isotropic margin of 2 mm.

The OARs were contoured for all patients, and they included the optic nerves, optic chiasm, brainstem, spinal cord, brain, retinas, corneas, lacrimal glands, lenses, cochleas and hippocampi [17]. The optic nerves, retinas, corneas, lacrimal glands, lenses and cochleas were divided into ipsilateral (I/L) and contralateral (C/L) in relation to the tumor site. A symmetric margin of 3 mm was added to the brainstem and spinal cord to generate the corresponding planning organ at risk volumes (PRVs).

### Dose prescription and planning objectives

The prescribed dose was 65 Gy in 26 fractions at 2.5 Gy per fraction. [18] The planning objective for all plans was that at least 98% of the CTV (V_95%_ ≥98%) receives 95% of the prescribed dose, at least 95% of the PTV (V_95%_ ≥95%) receives ≥95% of the prescribed dose and <2% of the PTV receives >107% of the prescribed dose (V_107%_ ≤2%). The planning objective for OARs included a D_0.5cc_ (the dose received by 0.5 ml of the volume) ≤69 Gy for the brain and V_7.2Gy_ (volume receiving a 7.2 Gy dose) ≤40% for the hippocampus [17, 19]. The planning objective for the neurological OARs (n-OARs) were a D_max_ (the maximum dose received by 0.1 ml of the volume of interest) ≤55 Gy for the optic nerve and chiasm, D_max_ ≤54 Gy for the brainstem and D_max_ ≤45 Gy for the spinal cord, as per the Quantitative Analyses of Normal Tissue Effects in the Clinic (QUANTEC) [20]. The planning objective for orbital structures (o-OARs) were a D_max_ ≤45 Gy for the retina, D_max_ ≤40 Gy for the cornea and lacrimal gland, and D_max_ ≤10 Gy for the lens [17, 21, 22]. For the cochlea, the objective was D_mean_ (the mean dose received by the volume) ≤35 Gy as per the QUANTEC.

Similar optimization constraints and planning parameters were used for the nc-IMRT plan, nc-VMAT plan and Hybrid IMRT/VMAT plan. The plans were optimized by enhancing CTV and PTV coverage as much as possible without exceeding the n-OAR constraints. First priority was given to sparing the n-OARs, and the other structures were given second priority.

### The nc-IMRT, nc-VMAT and Hybrid IMRT/VMAT treatment planning, beam configuration and optimization

Three plans were generated for each patient. All plans were generated using the RayStation v6.2 (RaySearch Laboratories, Stockholm, Sweden) treatment planning system with the collapsed cone convolution superposition (CCC)-based algorithm calculation by setting the dose grid to 0.2 × 0.2 × 0.2 cm^3^ and heterogeneity correction. All beam modeling was performed for the TrueBeam radiotherapy delivery system (Varian Medical System, Inc., Palo, Alto, CA, USA) equipped with a Millennium MLC that has 120 leaves. All plans were optimized by the trial and error method using uniform, Max, Min and EUD objective functions. A 5 cm auxiliary ring structure was created just outside and 2 cm from the PTV to help control dose spill beyond that limit, as shown in Supplementary Fig. 2. The nc-IMRT, nc-VMAT and Hybrid IMRT/VMAT plans were optimized by three experienced clinical physicists in consensus, and 40 iterations of all plans were performed for adequate plan optimization.

**Fig. 1.**
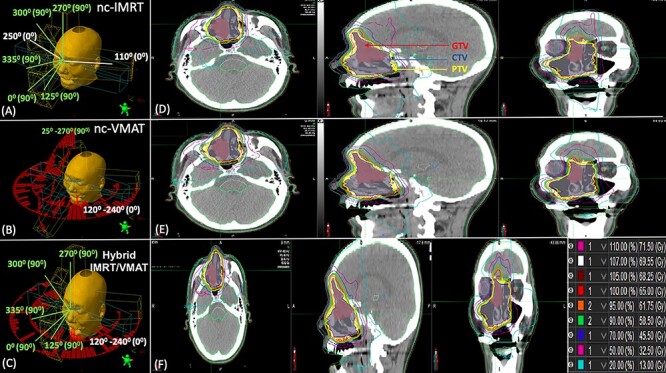
Overview of the beam configuration and dose distribution on axial, sagittal and coronal CT slices of the non-coplanar IMRT (A and D), non-coplanar VMAT (B and E) and Hybrid IMRT/VMAT (C and F) treatment plan. The beam direction range and couch angle in all patients is given per beam direction. The coplanar beam is marked in white, and the non-coplanar beam direction is marked in green.

The nc-IMRT (sliding window technique) plan was generated using a standard set of seven 6 MV beams, two coplanar beams (gantry at 110° and 250° with the couch at 0°), and five nc beams (gantry at 25, 0, 335, 300 and 270° with the couch at 90°), as shown in Fig. 1A. Direct aperture optimization was used, with the maximum number of segments restricted to 80, minimum segment area to 4 cm^2^ and minimum segment MU per fraction to 2.

The nc-VMAT plan was generated using four 6 MV arcs, two coplanar and two nc arcs, with the collimator rotated to 45° and 315°, to minimize the contribution of the tongue and groove effect. The angular extent for the coplanar arcs was 120 to 240° counterclockwise (CCW) and 240 to 120° clockwise (CW), and for the two nc arcs it was 25 to 270° CCW and 270 to 25° CW with the couch rotated to 90°, as shown in Fig. 1B. The maximum dose rate was 600 MU min^–1^ and the maximum leaf speed was 2.5 cm s^–1^.

The Hybrid IMRT/VMAT plan comprised five nc 6 MV IMRT (sliding window technique) beams and two 6 MV coplanar arcs. The beam arrangement of the five nc static fields was similar to the arrangement of the nc beams of the nc-IMRT plan. The angular extent of the two arcs was 120 to 240° CCW and 240 to 20° CW, as shown in Fig. 1C. The optimization algorithm optimized both the five nc beams IMRT and two arcs plan simultaneously. In the Hybrid IMRT/VMAT plan, the five nc IMRT beams were set to deliver half of the prescribed dose, and two arcs delivered the other half of the prescribed dose.

### Plan evaluation

The nc-IMRT, nc-VMAT and Hybrid IMRT/VMAT plans were evaluated by generating DVHs of the target (CTVs and PTVs) and OARs on nominal dose distributions. The D_2%_ (the dose received by 2% of the volume of interest), D_98%_ (the dose received by 98% of the target volume), median dose (the dose received by 50% of the target volume, D_50%_) and percentage of the CTV and PTV receiving 95% of the prescribed dose (V_95%_) were considered for CTV and PTV coverage. Dosimetric parameters for OARs were evaluated by D_max_ for n-OARs, retinas, corneas, lacrimal glands and lenses, D_mean_ for cochleas, D_0.5cc_ for the brain and V_7.2Gy_ for the hippocampi.

The quality of each plan was assessed by using the conformation number (CN) and homogeneity index (HI). The Radiation Therapy Oncology Group (RTOG) formula was used to calculate HI. Conformity around the CTV and PTV was determined by using the CN formula. The HI and CN are described using the following two equations:

(1) \begin{equation*} \mathrm{HI}=\frac{\mathrm{D}2_\%-\mathrm{D}98_\%}{\mathrm{D}50_\%} \end{equation*}

$\mathrm{CN}=\frac{{\Big(\mathrm{TV}95\Big)}^2}{\mathrm{TV}\times V95}$ (2)where TV95 is the target volume covered by 95% of the reference isodose, CTV and PTV are the target volumes (TVs) and V95 is the volume of 95% of the isodose.

The integral dose (Gy l^–1^) was computed to evaluate normal tissue sparing as shown in Equation 3.

(3) \begin{equation*} \mathrm{Integral}\ \mathrm{dose}=\mathrm{Dmean}\ \left(\mathrm{Gy}\right)\times \left(\mathrm{Body}\hbox{--} \mathrm{PTV}\right)\ \mathrm{volume}\ \mathrm{in}\ \mathrm{liters} \end{equation*}

where D_mean_ is average dose in Gy received by volume (Body – PTV) in liters. The computed MUs were assessed to identify treatment efficiency.

### EUD and NTCP evaluation

The digital imaging and communication in medicine (DICOM) standard radiotherapy doses from the nc-IMRT, nc-VMAT and Hybrid IMRT/VMAT plans were transferred to MIM (v6.86, MIM software Inc., Cleveland, OH, USA). Before calculating the EUD and NTCP, the linear-quadratic (LQ) equation with α/β of 2 (for the optic nerves and chiasm, brainstem and brain) was used to convert the cumulative dosage into an equivalent dose of 2 Gy (EQD2) per fraction as shown in Equation 4 [23].

(4) \begin{equation*} \mathrm{EQD}2=D\times \left(\frac{d+\frac{\upalpha}{\upbeta}}{2+\frac{\upalpha}{\upbeta}}\right) \end{equation*}

where ‘*D*’ is the total dose given in Gy, ‘*d*’ is dose per fraction and ‘α/β’ is the dose at which the linear and quadratic component of cell kill are equal. To estimate NTCP, the cumulative DVHs were converted into differential DVHs. NTCP was computed by log-logistic model using the generalized EUD concept (NTCP_Logit_) and Lyman–Kutcher–Burman models (NTCP_LKB_) [24–27]. According to this model, the NTCP_Logit_ and NTCP_LKB_ are described using the following five equations:

(5) \begin{equation*} {\mathrm{NTCP}}_{\mathrm{Logit}}=\frac{1}{1+{\left(\frac{{\mathrm{TD}}_{50}}{\mathrm{EUD}}\right)}^{4{\upgamma}_{50}}} \end{equation*}

(6) \begin{equation*} {\mathrm{NTCP}}_{\mathrm{LKB}}=\frac{1}{\sqrt{2\uppi}}{\int}_{-\infty}^t{\mathrm{e}}^{-\frac{t^2}{2}}\ \mathrm{d}x \end{equation*}

(7) \begin{equation*} t=\frac{\Big({D}_{\mathrm{eff}}-{\mathrm{TD}}_{50\Big)}}{\mathrm{m}{\mathrm{TD}}_{50}} \end{equation*}

(8) \begin{equation*} {D}_{e\mathrm{ff}}={{\left({\sum}_i{v}_i{D_i}^{\frac{1}{n}}\right)}_{\begin{array}{c}\ \\{}\ \end{array}}}^n \end{equation*}

(9) \begin{equation*} \mathrm{EUD}={{\left({\sum}_i{v}_i{D_i}^a\right)}_{\begin{array}{c}\ \\{}\ \end{array}}}^{\frac{1}{a}} \end{equation*}

where *D*_eff_ is identical to an EUD, and TD_50_ is the tolerance dose yielding a 50% complication rate of the normal organ. The parameters ‘m’ and ‘γ50’ represent the slope of the sigmoid dose–response curve, and the fractional volume of the organ is represented by ‘*v_i_*’ receiving a dose ‘*D_i_*’. The parameter ‘*n*’ describes the magnitude of volume effect, and (*D_i_*, *v_i_*) are the bins of differential DVH. The ‘*a*’ is a unitless parameter specific to the tumor or normal structure and is identified with the inverse of the volume effect parameter.

The RADBIOMOD Visual Basic for Application (VBA) software was used to compute EUD, NTCP_Logit_ and NTCP_LKB_ [28]. The parameters used for NTCP_Logit_ and NTCP_LKB_ are shown in Supplementary Table 1.

### Statistical analysis

The mean and standard deviation (SD) of all dosimetric parameters, EUD and absolute NTCP were computed for each plan cohort. The R commander EZR (v2.6-2) program (R software version 3.6.3) was used to perform all statistical calculations. The same CT scan of each patient was used to generate the IMRT, VMAT and Hybrid IMRT/VMAT plans. The repeated measure analysis of variance (ANOVA) test was used to compare the three techniques. The difference between the pair of technique was tested using Bonferroni post-hoc test. Two-tailed *P* values <0.05 were considered to be statistically significant.

## RESULTS

The patients’ characteristics are shown in Table 1. The median CTV and PTV values were 83.17 ml (range 40.16–260.21 ml) and 112.51 ml (range 56.74–314.28 ml), respectively.

**Table 1:** Patient characteristics

Patient	Age	Sex	Site	Location	Modified Kadish stage	Dulgerov stage	CTV volume (ml)	PTV volume (ml)
1	20 years	Male	NC	Right	C	T4	40.16	56.74
2	37 years	Female	NC	Right	C	T4	81.20	113.36
3	33 years	Female	NC	Left	C	T2	85.20	112.97
4	52 years	Female	NC	Left	C	T3	71.03	104.44
5	46 years	Female	NC	Left	C	T4	74.27	104.33
6	62 years	Female	NC	Left	C	T3	75.30	100.00
7	80 years	Male	NC	Right	C	T3	260.31	314.28
8	87 years	Female	NC	Left	C	T4	74.27	102.18
9	49 years	Female	NC	Right	C	T4	106.24	141.22
10	35 years	Male	NC	Right	C	T4	105.35	151.10
11	72 years	Male	NC	Right	C	T3	95.22	123.07
12	56 years	Male	NC	Right	C	T4	86.32	112.06

NC = nasal cavity; CTV = clinical target volume; PTV = planning target volume.

The nc-IMRT, nc-VMAT and Hybrid IMRT/VMAT plan dose distributions with isodose lines from 13 Gy (20%) to 71.50 Gy (110%) for a representative patient are shown in Fig. 1D–F. The CTV and PTV coverage goals for all patients were met with all three radiation modalities. The average cumulative DVHs for the CTV and PTV of a representative patient treated with the different plans are shown in Supplementary Fig. 3A and B. The mean values and SD of the study parameters for each of the three techniques were tabulated for the target (CTV and PTV) and OARs in Tables 2 and 3, and the radiobiological parameters (EUD, NTCP_Logit_ and NTCP_LKB_) in Table 4.

**Table 2:** CTV and PTV dosimetric parameters according to treatment techniques for all patients

Target structure	Dosimetric parameter	nc-IMRT^a^	nc-VMAT^b^	Hybrid IMRT/VMAT^c^	Pairwise comparison
CTV	D_2%_ (Gy)	69.36 ± 0.13	69.36 ± 0.17	69.10 ± 0.25	b versus c (0.001^*^) a versus c (<0.001^*^)
	D_98%_ (Gy)	64.72 ± 0.81	64.51 ± 0.89	64.73 ± 0.74	a versus b (0.10) b versus c (0.08)
	D_50%_ (Gy)	67.21 ± 0.16	67.37 ± 0.31	66.83 ± 0.49	b versus c (0.009^*^) a versus c (0.06)
	V_95%_ (%)	99.22 ± 0.62	99.33 ± 0.52	99.45 ± 0.45	NS^†^
	CN	0.55 ± 0.04	0.55 ± 0.05	0.57 ± 0.05	b versus c (0.006^*^)
	HI	0.07 ± 0.01	0.07 ± 0.01	0.06 ± 0.01	b versus c (0.002^*^) a versus c (<0.001^*^)
PTV	D_2%_ (Gy)	69.34 ± 0.14	69.34 ± 0.18	68.97 ± 0.25	b versus c (0.002^*^) a versus c (<0.001^*^)
	D_98%_ (Gy)	61.29 ± 1.48	61.44 ± 1.57	61.44 ± 1.61	NS^†^
	D_50%_ (Gy)	67.14 ± 0.23	67.22 ± 0.26	66.85 ± 0.20	b versus c (0.007^*^) a versus c (< 0.001^*^)
	V_95%_ (%)	97.88 ± 0.66	97.94 ± 0.71	97.96 ± 0.74	NS^†^
	V_107%_ (%)	1.02 ± 0.54	1.01 ± 0.56	0.52 ± 0.46	b versus c (0.026^*^) a versus c (0.001^*^)
	CN	0.72 ± 0.02	0.72 ± 0.05	0.74 ± 0.04	b versus c (0.034^*^)
	HI	0.12 ± 0.02	0.12 ± 0.02	0.11 ± 0.02	NS^†^

CTV = clinical target volume; PTV = planning treatment volume; D_(X%) =_ dose (Gy) received by (X%) of structure; V_(X% or X Gy)_ = percentage volume of structure receiving dose (X% or X Gy) or more; CN = conformation number; HI = homogeneity index; NS = non-significant

^*^Significant (*P* < 0.05).

^†^ *P* > 0.1.

**Table 3:** OAR dosimetric parameters according to treatment techniques for all patients

Target structure	Dosimetric parameter	nc-IMRT^a^	nc-VMAT^b^	Hybrid IMRT/VMAT^c^	Pairwise comparison *P* value
Spinal cord	D_max_ (Gy)	10.67 ± 4.06	10.2 ± 3.98	10.16 ± 3.69	NS^†^
Brainstem	D_max_ (Gy)	17.93 ± 11.60	17.70 ± 12.36	17.73 ± 12.53	NS^†^
Optic chiasm	D_max_ (Gy)	40.93 ± 9.31	39.63 ± 11.25	40.77 ± 9.03	NS^†^
I/L optic nerve	D_max_ (Gy)	54.18 ± 0.56	54.0 ± 0.61	53.86 ± 0.68	NS^†^
C/L optic nerve	D_max_ (Gy)	53.26 ± 2.63	52.25 ± 5.34	51.52 ± 4.37	a versus c (0.042^*^)
I/L retina	D_max_ (Gy)	43.17 ± 3.10	43.02 ± 2.69	42.35 ± 2.64	NS^†^
C/L retina	D_max_ (Gy)	40.42 ± 6.02	37.49 ± 8.66	37. 25 ± 8.07	NS^†^
I/L cornea	D_max_ (Gy)	19.44 ± 6.32	17.80 ± 5.78	16.04 ± 5.27	a versus c (0.055)
C/L cornea	D_max_ (Gy)	14.13 ± 5.56	12.21 ± 4.00	11.76 ± 2.85	NS^†^
I/L lens	D_max_ (Gy)	8.45 ± 1.58	8.29 ± 1.67	7.84 ± 1.15	NS^†^
C/L lens	D_max_ (Gy)	8.17 ± 1.81	7.54 ± 1.35	7.39 ± 1.62	a versus c (0.08)
I/L lacrimal gland	D_max_ (Gy)	12.69 ± 4.80	12.92 ± 6.95	12.50 ± 6.52	NS^†^
C/L lacrimal gland	D_max_ (Gy)	10.73 ± 2.21	12.09 ± 3.70	10.19 ± 2.97	b versus c (0.021^*^)
I/L cochlea	D_mean_ (Gy)	14.53 ± 7.58	15.90 ± 7.35	14.53 ± 6.74	b versus c (0.020^*^)
C/L cochlea	D_mean_ (Gy)	9.67 ± 7.83	11.19 ± 7.02	10.31 ± 6.36	a versus b (0.099)
Brain	D_0.5cc_ (Gy)	66.20 ± 1.38	66.07 ± 1.37	65.67 ± 1.80	NS^†^
Hippocampus	V_7.2Gy_ (%)	24.02 ± 8.74	23.84 ± 9.21	19.66 ± 11.61	NS^†^
Integral dose	Gy l^–1^	49.88 ± 10.73	49.31 ± 12.22	48.40 ± 11.97	NS^†^
Monitor units	MUs	752.13 ± 48.11	565.89 ± 88.97	744.49 ± 127.44	a versus b (<0.001^*^) b versus c (<0.001^*^)
Treatment time	Min	1.25 ± 0.08	2.16 ± 0.02	2.04 ± 0.15	a versus b (<0.001^*^) a versus c (0.002^*^)

OAR = organs at risk; D_(X% or cc) =_ dose (Gy) received by (X% or X ml) of structure; V_(X% or XGy)_ = percentage volume of structure receiving dose (X% or X Gy) or more; I/L = ipsilateral; C/L = contralateral; NS = non-significant; D_max_ = the maximum dose reported as the dose received by 0 ml of the volume of interest; D_mean_ = the mean dose received by the volume of interest.

^*^Significant (*P* < 0.05).

^†^ *P* > 0.1.

**Table 4:** The average EUD and NTCP values for optic nerves, optic chiasm, brainstem and brain

Organ at risk Structure	EUD and NTCP	nc-IMRT^a^	nc-VMAT^b^	Hybrid IMRT/VMAT^c^	Pairwise comparison *P* value
I/L optic nerve	EUD (Gy)	44.06 ± 4.96	44.91 ± 3.69	42.23 ± 5.48	b versus c (0.015^*^) a versus c (0.042^*^)
	NTCP_Logit_ (%)	1.63 ± 1.72	1.66 ± 1.45	1.24 ± 1.52	b versus c (0.021^*^) a versus c (0.029^*^)
	NTCP_LKB_ (%)	0.07 ± 0.12	0.09 ± 0.10	0.05 ± 0.08	NS^†^
C/L optic nerve	EUD (Gy)	39.56 ± 5.99	39.90 ± 9.29	37.08 ± 9.18	b versus c (0.034^*^) a versus c (0.021^*^)
	NTCP_Logit_ (%)	0.63 ± 0.78	0.91 ± 1.11	0.60 ± 0.98	b versus c (0.032^*^)
	NTCP_LKB_ (%)	0.01 ± 0.02	0.03 ± 0.09	0.02 ± 0.07	NS^†^
Optic chiasm	EUD (Gy)	24.42 ± 9.70	21.35 ± 10.42	23.04 ± 8.89	a versus b (0.041^*^)
	NTCP_Logit_ (%)	0.04 + 0.08	0.02 ± 0.03	0.03 ± 0.10	NS^†^
	NTCP_LKB_ (%)	0.003 ± 0.00	0.00 ± 0.00	0.00 ± 0.00	NS^†^
Brainstem	EUD (Gy)	3.98 ± 4.58	4.67 + 5.51	4.14 ± 4.56	NS^†^
	NTCP_Logit_ (%)	0.00 ± 0.00	0.00 ± 0.00	0.00 ± 0.00	NS^†^
	NTCP_LKB_ (%)	0.00 ± 0.00	0.00 ± 0.00	0.00 ± 0.00	NS^†^
Brain	EUD (Gy)	26.51 ± 5.08	25.52 ± 3.71	24.99 ± 3.74	NS^†^
	NTCP_Logit_ (%)	0.00 ± 0.00	0.00 ± 0.00	0.00 ± 0.00	NS^†^
	NTCP_LKB_ (%)	0.00 ± 0.00	0.00 ± 0.00	0.00 ± 0.00	NS^†^

nc-IMRT = non-coplanar IMRT; nc-VMAT = non-coplanar VMAT; NTCP = normal tissue complication probability; EUD = equivalent uniform dose; LKB = Lyman–Kutcher–Burman model; I/L = ipsilateral; C/L = contralateral; NS = non-significant.

^*^Significant (*P* < 0.05).

^†^ *P* > 0.01.

### Target dosimetric parameters

The Hybrid IMRT/VMAT plan has no significant difference in terms of V_95%_ coverage for the CTV and PTV in comparison with the nc-IMRT and nc-VMAT plan (*P* ≥ 0.05). Significantly better CNs for CTV and PTV were obtained with the Hybrid IMRT/VMAT plan than with the nc-VMAT plan (0.57 versus 0.55, *P* = 0.006; 0.74 versus 0.72, *P* = 0.034). The HI for CTV was significantly better with the Hybrid IMRT/VMAT plan because of the higher D_2%_ obtained with the nc-IMRT plan and nc-VMAT plan (Table 2; Supplementary Fig. 4). The mean V_107%_ with the Hybrid IMRT/VMAT plan was 0.54%, with a difference of 0.50% lower than for the nc-IMRT (*P* ≤ 0.001) and 0.49% lower than for the nc-VMAT (*P* = 0.026).

### Organs at risk

Hybrid IMRT/VMAT plans shows equivalent sparing of OARs in comparison with nc-IMRT and nc-VMAT, but the Hybrid IMRT/VMAT plan achieved a better (*P* ≤ 0.05) result for C/L optic nerve, C/L lacrimal gland and I/L cochlea. D_max_ obtained using the Hybrid IMRT/VMAT plan to the C/L optic nerve was 51.52 Gy, and was 1.74 Gy lower than with the nc-IMRT plan (*P* = 0.042).

### Integral dose, MU delivered and beam-on time

The MUs delivered per fraction were significantly higher with the nc-IMRT plan and Hybrid IMRT/VMAT in comparison with the nc-VMAT plan [increase of 187 MU (*P* ≤ 0.001] and 179 MU (*P* ≤ 0.001), respectively), but there were no significant differences in their integral doses. The Hybrid IMRT/VMAT plan average beam-on time was 2.04 min, and was slightly shorter than that of nc-VMAT by 7 s, but this was statistically non-significant. The nc-IMRT plan gave the shortest beam-on treatment time, with an average of 1.25 min.

### EUD and NTCP

The mean EUD obtained for the I/L optic nerve with the Hybrid IMRT/VMAT plan was 42.23 Gy, 1.83 Gy lower than with the nc-IMRT plan (*P* = 0.042) and 2.68 Gy lower than with the nc-VMAT (*P* = 0.015) plan. For the C/L optic nerve with the Hybrid IMRT/VMAT plan, the mean EUD was 37.08 Gy, which was 2.82 Gy lower than with the nc-VMAT plan (*P* = 0.034) and 2.48Gy lower than with the nc-IMRT plan (*P* = 0.021). The mean EUD of the optic chiasm with nc-VMAT was 21.35 Gy, and 3.07 Gy lower than with nc-IMRT (*P* = 0.041). The Hybrid IMRT/VMAT plan yielded an NTCP_Logit_ difference of <1% for optic nerves in comparison with nc-IMRT and nc-VMAT. The NTCP_LKB_ for the n-OARs and brain were similar with all three plans (*P* > 0.05).

## DISCUSSION

Potential clinical outcome advantages for most sinonasal cancers, including in their unresectable stages, have been achieved by using nc-IMRT and nc-VMAT plans in comparison with coplanar IMRT and VMAT plans [13, 29]. Studies by Wiegner *et al*. and Dirix *et al*. reported that new and technical advancements in radiation delivery would be needed to improve the outcomes of sinonasal cancer patients by strengthening tumor coverage and by decreasing late toxicity by sparing OARs [30, 31]. These observation led to the evaluation of the Hybrid IMRT/VMAT treatment strategy as previously reported in relation to esophageal cancer and brain metastasis [32, 33].

Our study compared a Hybrid IMRT/VMAT plan with an nc-IMRT and an nc-VMAT plan in patients with unresectable ONB located close to surrounding n-OARs and o-OARs. The results of the present study showed that the Hybrid IMRT/VMAT technique provided an overall dosimetric advantage over nc-VMAT in regard to the conformity of the CTV and PTV, which is important when the tumor is in close proximity to n-OARs that impede adequate PTV coverage. Hybrid IMRT/VMAT significantly reduced the D_max_ of the C/L optic nerve compared with nc-IMRT; this decrease in D_max_ can be clinically correlated by a decline in the radiobiological parameters EUD and NTCP_Logit_.

Several studies in regard to sinonasal cancer have shown the superiority of different planning techniques using DVH-specific point parameters, but whether the treatment plan superiority can be translated into a clinical benefit is unclear [13, 14]. In contrast to these studies, we performed EUD and NTCP assessments in regard to radiation-related neurological toxicity to the optic nerves, optic chiasm, brainstem and brain. The results of our study showed that the Hybrid IMRT/VMAT plan yielded lower EUDs to the I/L and C/L optic nerve in comparison with the nc-IMRT plan and nc-VMAT plan. A total cumulative fractionated radiation dose of >50 Gy may produce radiation-induced optic neuropathy (RION) with a peak incidence between 10 and 20 months and a 10-year actuarial risk of up to 5% for a dose of 50–60 Gy [34]. In the study by Moiseenko *et al*., comparing dose–response characteristics of four NTCP models using outcomes for RION, the NTCP differences between models were <1%, and the differences in these values predicted by different models were <1.5 Gy [35]. This result is in accord with our study, and the clinical benefit of these small differences in dosimetric data, EUD and NTCP of optic nerves reported in our study may be minimal.

Because a significant dosimetric advantage had been reported when two or more arcs were used to treat sinonasal cancers, in our study we used two arcs in the Hybrid IMRT/VMAT plan and four arcs in the nc-VMAT plan [12, 13]. Since an increase in the modulation freedom had been observed by adding one or more arcs in the sagittal plane, we used two sagittal arcs in the nc-VMAT plan [13]. Because of the complexity of the target, nine-field IMRT was basically used to treat the patients. Since nine-field IMRT involves a longer treatment delivery time and more MUs that can cause an increase in the risk of intrafraction target movements and the risk of a secondary malignancy, we used seven beams in the nc-IMRT plan and five beams in the Hybrid IMRT/VMAT plan [36]. The Hybrid IMRT/VMAT technique was found to be no more difficult during the planning steps than standard inverse IMRT and VMAT. It might reduce the learning curve for planning for dosimetrists and physicists with previous experience using RayStation software.

This study had several limitations. We did not assess the impact of the prescription dose ratio between IMRT and VMAT in the Hybrid IMRT/VMAT plan on dose distribution and delivery efficiency by changing the weighting parameters to 1:2 or 2:1. A general limitation of this planning study was that the results might not be consistent when the calculation or optimization algorithm, beam parameter arrangement, and target and OAR constraints in multiobjective optimization are changed. However, there will always be differences between absolute and relative plan quality because of the inherent differences between inverse planning strategies and algorithms throughout clinical and research optimization systems. NTCP-based plan ranking can be model dependent, and no model can be deemed to be a preferred model. For radiation-induced optic neuropathy, large variations in model parameters may be observed between the models [35]. Thus, cautious interpretation of the results of this study is essential. The sample size of 12 patients in this study was small, but an attempt was made to reduce this bias by using paired data statistics to increase test sensitivity for possible differences between the plans.

In future studies, the ideal beam orientation for the Hybrid IMRT/VMAT plan would be likely to differ for different sites; further research will be necessary to identify the cancer sites and geometries that will benefit most from the Hybrid IMRT/VMAT technique. Without switching between the current separate IMRT and VMAT components, the IMRT and VMAT technique (FusionArc technique) could be delivered as a modulated arc with IMRT control points within the VMAT control point sequence, with a decrease in treatment delivery time.

In conclusion, the Hybrid IMRT/VMAT technique, when treating unresectable ONB, can provide better dose distributions by improving target conformity. The Hybrid IMRT/VMAT plan significantly reduces D_max_ to the C/L optic nerve compared with the nc-IMRT plan. Also, EUDs for both optic nerves were reduced compared with nc-IMRT and nc-VMAT. Hybrid IMRT/VMAT can therefore be considered as a viable treatment technique for unresectable olfactory neuroblastoma. However, the clinical benefit of these small differences in dosimetric data, EUD and NTCP_Logit_ (<1%) of optic nerves reported in our study may be minimal.

## Funding

This work was supported in part by the ‘Japan Agency for Medical Research and Development’ (AMED) [18ck0106210h0003, 19ck0106485h001] and the ‘National Cancer Center Research and Development Fund’ [31-A-17].

## Conflict of Interest 

All authors declares that they have no conflict of interest.

## Supplementary Material

Supplementary_Figure_1_rrab010

Supplementary_Figure_2_rrab010

Revised_Supplementary_Figure_3_rrab010

Revised_Supplementary_Figure_4_rrab010

Revised_Supplementary_Table_1_rrab010
