# Supplementary figures and images for: Comparison of a Hybrid IMRT/VMAT technique with non-coplanar VMAT and non-coplanar IMRT for unresectable olfactory neuroblastoma using the RayStation treatment planning system—EUD, NTCP and planning study
Source: J Radiat Res. 2021 Apr 12;62(3):540–8. doi: 10.1093/jrr/rrab010 (PMC8127663; doi:10.1093/jrr/rrab010)

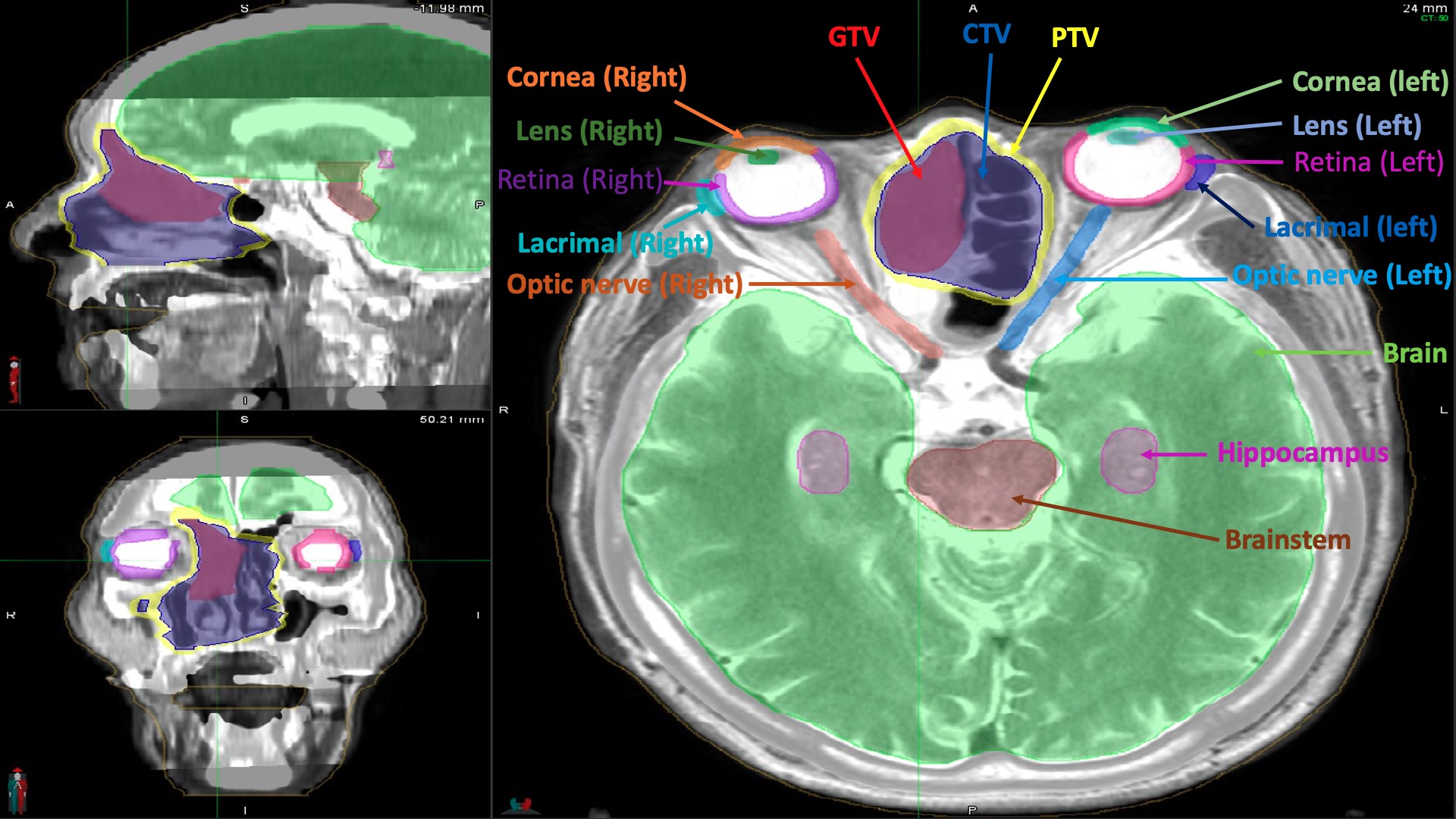

Supplement: Supplementary_Figure_1_rrab010 [file supplementary_figure_1_rrab010.jpeg]

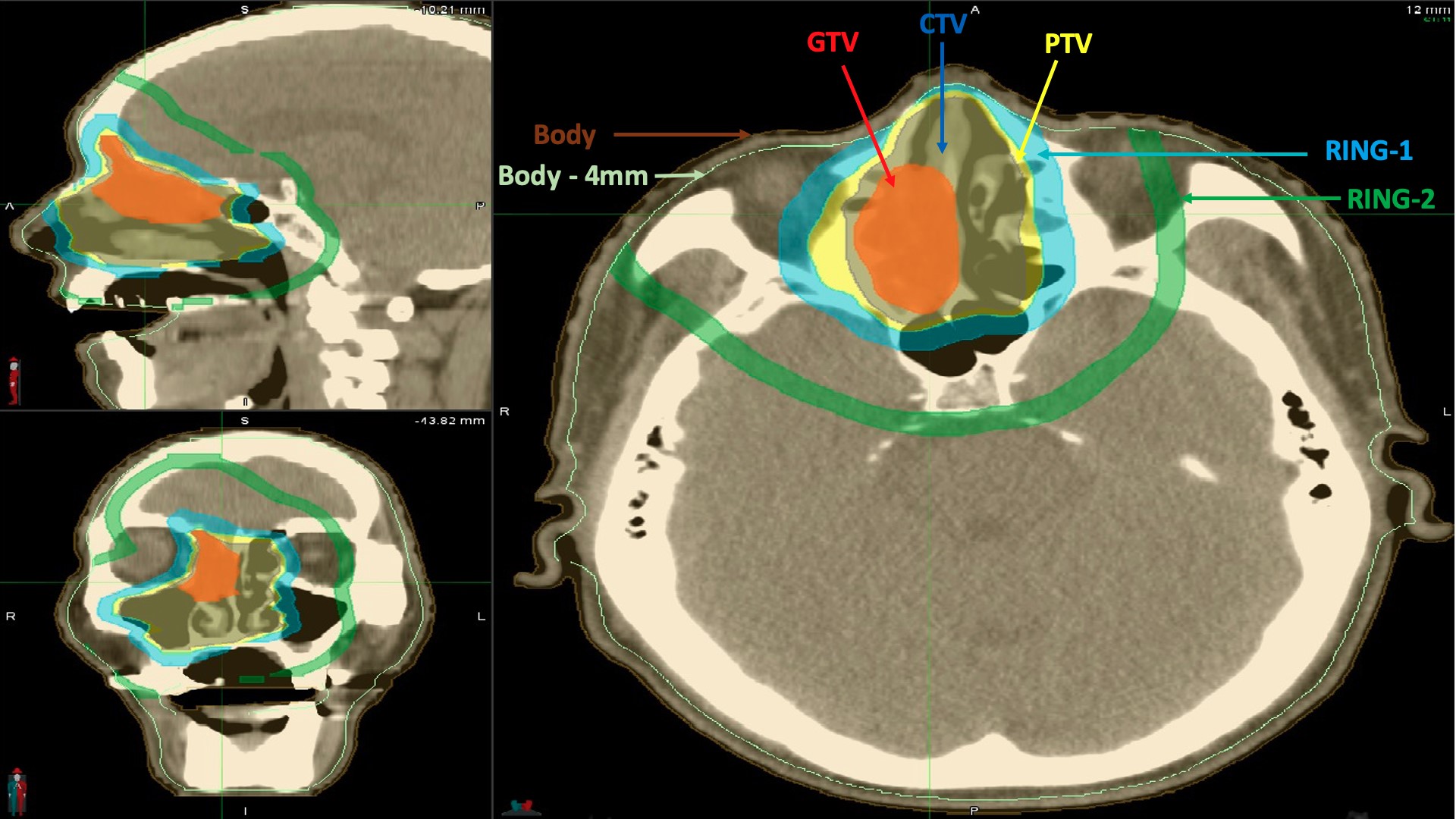

Supplement: Supplementary_Figure_2_rrab010 [file supplementary_figure_2_rrab010.jpeg]

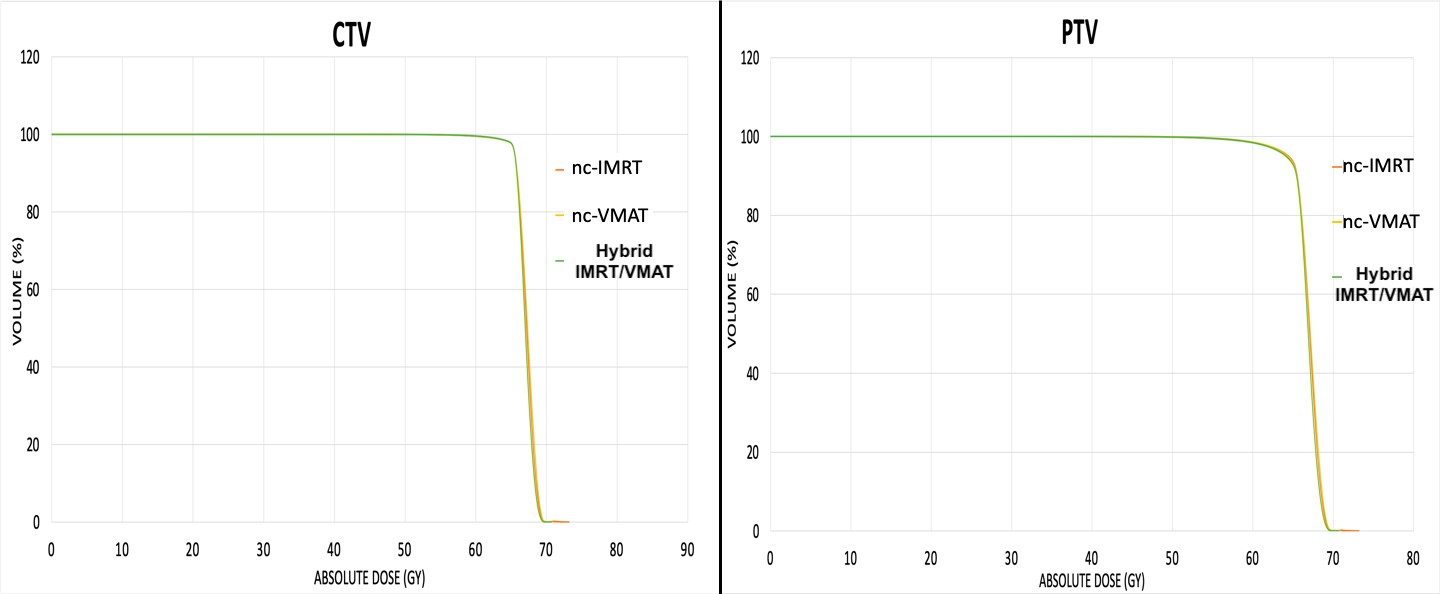

Supplement: Revised_Supplementary_Figure_3_rrab010 [file revised_supplementary_figure_3_rrab010.jpeg]

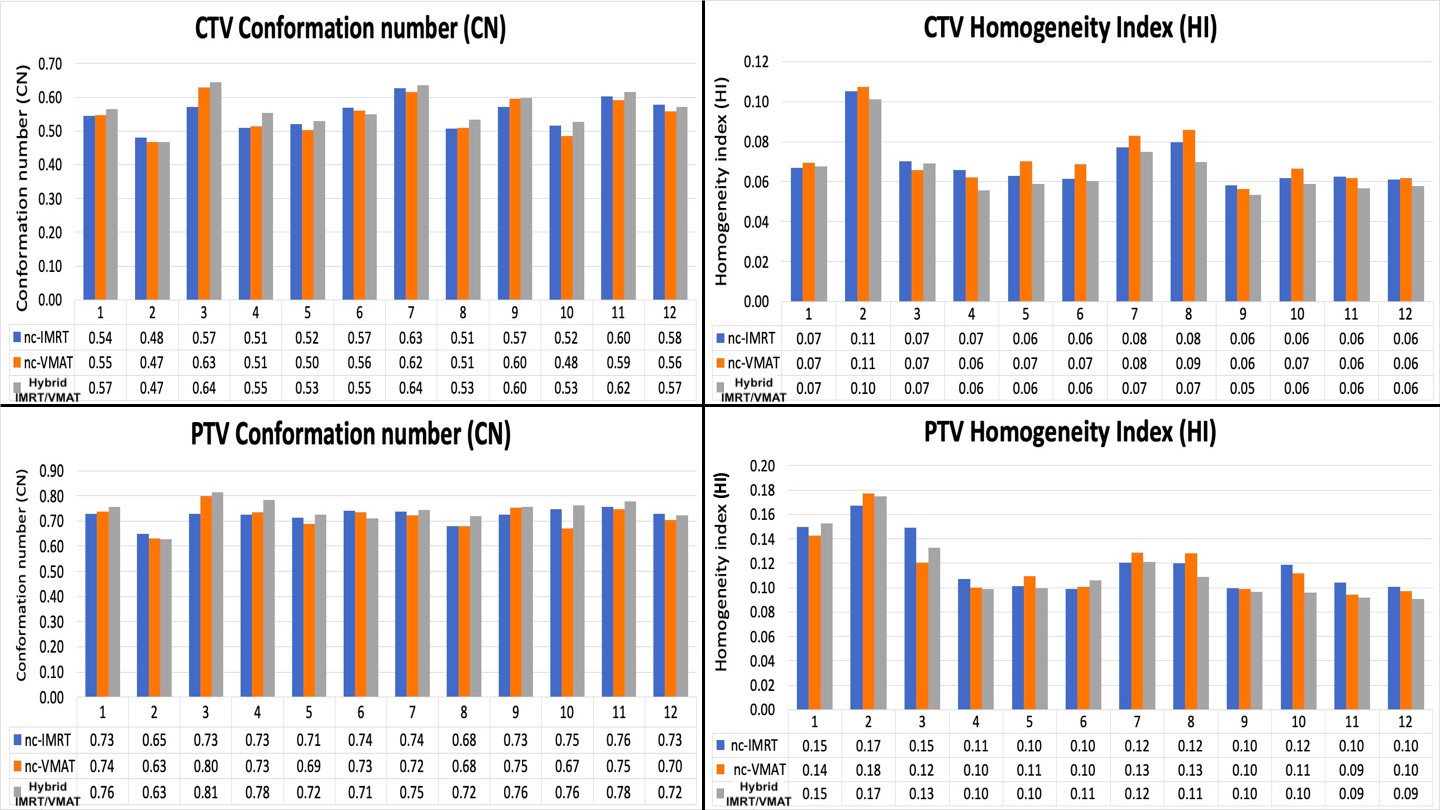

Supplement: Revised_Supplementary_Figure_4_rrab010 [file revised_supplementary_figure_4_rrab010.jpeg]
