# Supplementary material for: Comparison of a Hybrid IMRT/VMAT technique with non-coplanar VMAT and non-coplanar IMRT for unresectable olfactory neuroblastoma using the RayStation treatment planning system—EUD, NTCP and planning study
Source: J Radiat Res. 2021 Apr 12;62(3):540–8. doi: 10.1093/jrr/rrab010 (PMC8127663; doi:10.1093/jrr/rrab010)
Supplement: Revised_Supplementary_Table_1_rrab010 [file revised_supplementary_table_1_rrab010.docx]

Supplementary Table 1: Radiobiological parameters for NTCP models

| OAR Structure | NTCP | Radiobiological Parameters |
| --- | --- | --- |
| Optic nerve and chiasm | NTCP_Logit_ | TD_50 (Gy)_ = 65, a = 25, γ_50_ = 3 |
|  | NTCP_LKB_ | TD_50 (Gy)_ = 65, n = 0.25, m = 0.14 |
| Brainstem | NTCP_Logit_ | TD_50 (Gy)_ = 65, a = 7, γ_50_ = 3 |
|  | NTCP_LKB_ | TD_50 (Gy)_ = 65, n = 0.16, m = 0.14 |
| Brain | NTCP_Logit_ | TD_50 (Gy)_ = 60, a = 5, γ_50_ = 3 |
|  | NTCP_LKB_ | TD_50 (Gy)_ = 60, n = 0.25, m = 0.15 |

Abbreviation: NTCP = normal tissue complication probability; EUD = equivalent uniform dose; LKB = Lyman-Kutcher-Burman model; TD_50_ (Gy) = dose at which there is 50% chance of complication of the normal organ; γ_50_ = slope of dose-response curve for NTCP_Logit_; m = slope of dose-response curve for NTCP_LKB_; n = dose-volume relationship, a = tissue specific parameter that describes the volume effect
